# Numerous News Items

**Published:** 1894-04

**Authors:** 


### Medical News and Miscellany.

---

**That splendid paper** promised the JOURNAL by Hon. D. G. Wooten, and announced in our last, was not forthcoming. Sorry.

---

**U. S. Pension Examiner.**—Dr. T. J. Bennett has been apppointed Medical Examiner on the Pension Board at Austin, vice Dr. F. E. Daniel, resigned.

**An Electric Battery**—new—for sale by this journal at a reduction.

---

**Wanted**—To hear from every Texas Doctor who wants to sell his property and change location. Address THE JOURNAL.

---

**The Fourth Annual Meeting** of the Association of Military Surgeons of the United States will be held at Washington, D. C., May 1st, 2d and 3d, 1894.

---

**Handy for Physicians.**—The J. Singer Book Co., of Galveston, now represent the great publishing house of P. Blakiston, Son & Co., Philadelphia, and are prepared to furnish the Texas profession all the medical publications from that house.

---

**Waco.**—The little outbreak of scarlet fever at Waco was promptly suppressed. The State Health Officer visited Waco and found the health officer there vigilant and efficient, and the few cases that had occurred were promptly isolated.

---

**The senior** takes opportunity, unbeknown'st to his modest junior, to announce the advent, since last issue, of a son, born to Dr. and Mrs. S. E. Hudson, and to extend to the happy parents warmest congratulations. He is a bouncing boy, and—just like his handsome *papa*.

---

**Drs. Brown and Baily,** in this issue, submit the history of a man who died some sixty or eighty days after having been bitten by a puppy, and ask if it were hydrophobia. Criticisms by our readers are invited. Our own opinion is that it was not hydrophobia; the absence of the characteristic convulsive features would seem to be sufficient to exclude hydrophobia.

---

**Doctor:** In making up your estimate of expenses for the trip to Austin to the big State Medical Convention, slip in an extra $2 blll for the JOURNAL,—those of you who are not subscribers, you will need it in your business. Subscribers are requested to do the same for renewal, or for arrears. Some few (who have unintentionally neglected us) are invited to put in *two* little $2 bills, to help get shoes and stockings for Bettie and the baby.

---

**Dr. T. J. Tyner,** of Austin, and his estimably lady, sailed from New York on the 18th March for Rome, where the doctor was appointed to read at the International Congress, on 29th March, a paper on his operation for cataract. He is the originator of a preliminary capsulotomy, a step whereby the extraction of the cataract is much simplified and the after-treatment much shortened. Dr. and Mrs. Tyner will return to Austin by the 20th of April inst., in time for the doctor to attend the meeting of the Texas State Medical Association.

---

**Neuralgic Dysmenorrhea.**—In the *Rivista Clinica e Terapeutica*, November 12, 1893, the following formula is praised:

Extract of opium, }
Extract of belladonna, } aa cgms. 10
(grs. jss).
Sulphate of quinine.......... gm. 1
(grs. xv).

Sufficient for twenty-five pills. One every three hours.

—*Pritchard.*

---

"**Down in Texas.**—Editor Daniel, of the TEXAS MEDICAL JOURNAL, keeps up his record for publishing a red-hot journal in red.

"It is an uncommon day when he does not pitch into some one, or make an effort to reform something."—*Medical Sentinel, Portland, Oregon.*

Right you are, brother *Sentinel.* There is so much that needs pitching into, and so much that needs reforming, that we fee we are neglecting our duty unless we do take a hand.

---

**Here's Your Chance, Doctor.**—Make up your list of journals for 1894 from the following:

| | |
|---|---|
| TEXAS MEDICAL JOURNAL ($2.00) will be sent in connection with the JOURNAL AMERICAN MEDICAL ASS. ($5.00 a year), both for | $6 00 |
| Annals Gynecology and Pediatry ($2.00), both for | 3 60 |
| Annals Hygiene ($2.00), both for | 3 60 |
| Annals Surgery ($5.00), both for | 6 00 |
| International Journal Surgery ($1.00) | 2 75 |
| University Medical Magazine ($2.00) | 3 60 |
| Texas Medical Journal and "Cosmopolitan," both for | 3 25 |

---

**Mr. W. H. Holland,**, Superintendent of the Texas (colored) Deaf and Dumb and Blind Institute, authorizes the JOURNAL to extend to visiting physicians and delegates to the Texas State Medical Association a cordial invitation to visit the Institute during their stay in Austin, and will be pleased to extend to them every courtesy in his power. This institution is a feature of Texas, great charity of which the State may well be proud, and we are informed by the Board of Managers that it is managed very creditably by the present Superintendent, and a visit will repay any physician who feels an interest—as all must do—in the care of our unfortunates.

---

**Symmetrical Lipoma.**—Dr. J. P. Coffy, of Plano, Texas, writes the JOURNAL that some years ago a New York college professor exhibited to the class a case of symmetrical lipoma, and stated that such cases are extremely rare. Dr. Coffy says he wants Texas to keep up with the procession, and he reports to the JOURNAL the case of a man, who, nine years ago, worked on a farm and weighed 165 pounds. He quit farming, engaging in mercantile business; and within twelve months his weight increased to 235 lbs., and he developed a series of fatty tumors, equal in number and about equal size, on each arm and each side of the chest. The tumors will average 1x1½ inches in diameter, and have not increased in size lately.

---

**While the doctors** are here at the great annual meeting of the State Medical Association, it will be worth their while to make a trip out to the Confederate Home. Capt. Barnette, the Superintendent, authorizes the JOURNAL to extend to them a cordial invitation to do so. They may, no doubt will, the younger ones particularly, feel an interest in beholding these hoary ruins of a former generation,—grim and gray relicts of a glorious, none the less glorious because lost, cause; and reflect that when the dread alarum sounded that waked the country to war, these men sprang to arms from homes as sweet and peace as serene as that the younger ones now know: and that then, to these men, life looked as rosy and inviting, as full of hope and promise, as it now appears to the young. They sacrificed all but honor in the cause they believed to be right. The State has done nobly in providing this Home, where these heroes may be sheltered and provided for, in their last days, from the sufferings and infirmities that come with old age. They did their best; mortal man could have done no more.

---

**A Question of Veracity.**—The *Lancet-Clinic* of March 24th, copies entire, Mr. Ryder-Taylor's letter in our last issue, "Do Newspapers Foster Abortion," and our editorial comments thereon, and says:

"The above letter and editorial are from the current issue of the TEXAS MEDICAL JOURNAL, both of which at this particular time, are of interest to the medical profession.

"For Mr. Ryder-Taylor's information we have to say that Mr. George E. Gurrier, the well-known telegraphic correspondent of New York, has not denied the authenticity of the fake 'ads.' of the quack Amick Company sent out by him, nor has he instituted any libel suit against the Cincinnati *Lancet Clinic* for the exposure of that infamous business, which was deliberately entered into and carried out for the intentional deception of the people.

"We have in our possession Mr. George E. Gurrier's order to the Louisville *Anzeiger*. Our hand is right in the libel suit business, and we would just as leave as not have Mr. Gurrier proceed against us at once, as he is one of the connecting links in the little chain of that affair. His attention is directed to the little weeping episode of his confrère, published on another page of this issue of the *Lancet-Clinic*, with a suggestion that he at once hasten to his relief. He will thereby gain a little new experience before opening up his own smooth-bore batteries."

